# Oxidative Stress and Skin Fibrosis

**DOI:** 10.1007/s40139-014-0062-y

**Published:** 2014-10-17

**Authors:** Anjali Shroff, Andrew Mamalis, Jared Jagdeo

**Affiliations:** 1 Department of Dermatology, Icahn School of Medicine at Mount Sinai, Clinical Research – Dermatology, 5 East 98th Street- 5th floor, Box 1048, New York, NY USA; 2 Department of Dermatology, University of California Davis, Sacramento, CA USA; 3 Dermatology Service, Sacramento VA Medical Center, Mather, CA USA; 4 Department of Dermatology, State University of New York Downstate Medical Center, Brooklyn, NY USA

**Keywords:** Skin fibrosis, Skin scarring, Scars, Oxidative stress, Free radicals, Therapy

## Abstract

Fibrosis is defined as increased fibroblast proliferation and deposition of extracellular matrix components with potential clinical ramifications including organ dysfunction and failure. Fibrosis is a characteristic finding of various skin diseases which can have life-threatening consequences. These implications call for research into this topic as only a few treatments targeting fibrosis are available. In this review, we discuss oxidative stress and its role in skin fibrosis. Recent studies have implicated the importance of oxidative stress in a variety of cellular pathways directly and indirectly involved in the pathogenesis of skin fibrosis. The cellular pathways by which oxidative stress affects specific fibrotic skin disorders are also reviewed. Finally, we also describe various therapeutic approaches specifically targeting oxidative stress to prevent skin fibrosis. We believe oxidative stress is a relevant target, and understanding the role of oxidative stress in skin fibrosis will enhance knowledge of fibrotic skin diseases and potentially produce targeted therapeutic options.

## Introduction

Fibrosis is caused by increased tissue remodeling interrupting normal function and is a common cellular response to long-term inflammation or cell injury [1••]. Though usually a beneficial tissue response, increased fibrosis can lead to organ dysfunction and degenerative changes in vascular illnesses such as diabetes, hypertension, and chronic kidney disease [2]. The effects of fibrosis are prominent in dermatological diseases such as scleroderma, graft-versus-host disease (GVHD), keloids, and other fibrotic diseases. Fibrotic skin disorders can greatly impact patient quality of life with organ dysfunction and psychological sequelae [3••, 4–6]. Despite such serious effects, mechanisms of fibrosis are still not completely understood, and the need for anti-fibrotic treatments remains [3••].

Fibrosis is initiated by cellular injury due to prolonged injury, inflammation, infection, autoimmune reactions, allergy, radiation, or chemical damage [7, 8]. Subsequent activation of inflammatory cells, elevations of oxidative stress, uncontrolled increase in fibroblast number, and deposition of extracellular matrix (ECM) components characterizes fibrosis. Oxidative stress, an imbalance of oxygen- and nitrogen-based free radical production and the cellular antioxidant defense system, has an important role in the pathogenesis of fibrosis with effects on cellular pathways of function and repair [3••, 9]. Consequently, there has been increasing research into the role of oxidative stress in fibrotic skin disease.

In particular, therapeutic modalities targeting these radicals in fibrotic diseases have been of special interest. To our knowledge, there are no published reviews that primarily focus on the role of oxidative stress in skin fibrosis. In this review, we discuss the mechanisms via oxidative stress that promote skin fibrosis highlighting specific fibrotic skin disorders. We also seek to expound on a variety of potential therapeutics—particularly focusing on cellular targets and mechanisms of action.

## Methods

A search of the published literature from 1 January 2009 to present on the role of oxidative stress in skin fibrosis was performed in July 2014. The following fibrotic skin disorders were identified after review of the textbook *Dermatology* [10•]: acral fibrokeratoma, amyloidosis, atypical fibroxanthoma, bleomycin-induced skin fibrosis, cutaneous angiofibroma, dermatofibroma, dermatofibroma protuberans, eosinophilia–myalgia syndrome (EMS), eosinophilic fasciitis, epithelioid cell histiocytoma, epithelioid sarcoma, fibroblastic rheumatism, fibroma of the tendon sheath, fibrosarcoma, fibrous hamartoma, graft-versus-host disease, hypertropic scars, infantile digital fibroma, infantile myofibromatosis, keloids, lipodermatosclerosis, mixed connective tissue disease, multinucleate cell angiohistiocytoma, nephrogenic systemic fibrosis, nodular fasciitis, porphyria cutanea tarda, restrictive dermopathy, scleredema, scleredema diabeticorum, scleroderma, scleromyxedema, sclerotic fibroma of the skin, stiff skin syndrome, superficial fascial fibromatosis, taxane-induced skin fibrosis, toxic oil syndrome (TOS), and Winchester syndrome. A search of PubMed and EMBASE was conducted using specific keywords or MeSH terms. “Fibrosis” was combined with (“oxidative stress,” “reactive nitrogen species,” or “reactive oxygen species”) along with the above listed disorders. Papers published within the last 5 years were included. Papers in a language other than English were excluded. Additional articles were included from the bibliography of articles meeting the search criteria.

## Results

As outlined in Table 1, our initial search resulted in 131 articles from the Pubmed database. A total of 131 articles were considered and screened. A title and abstract screen was conducted, exclusion of articles not in English was completed, and duplicates removed with a total of 54 articles remaining. Additional articles from the bibliography of the articles meeting the search criteria were included with a total of 57 articles in this review.

**Table 1:**
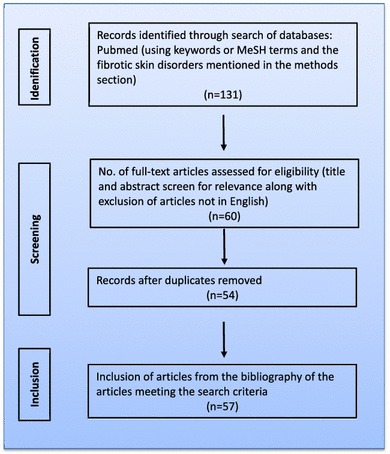
Schematic of literature search strategy and results

## The Role of Oxidative Stress in Skin Fibrosis

**Fig. 1:**
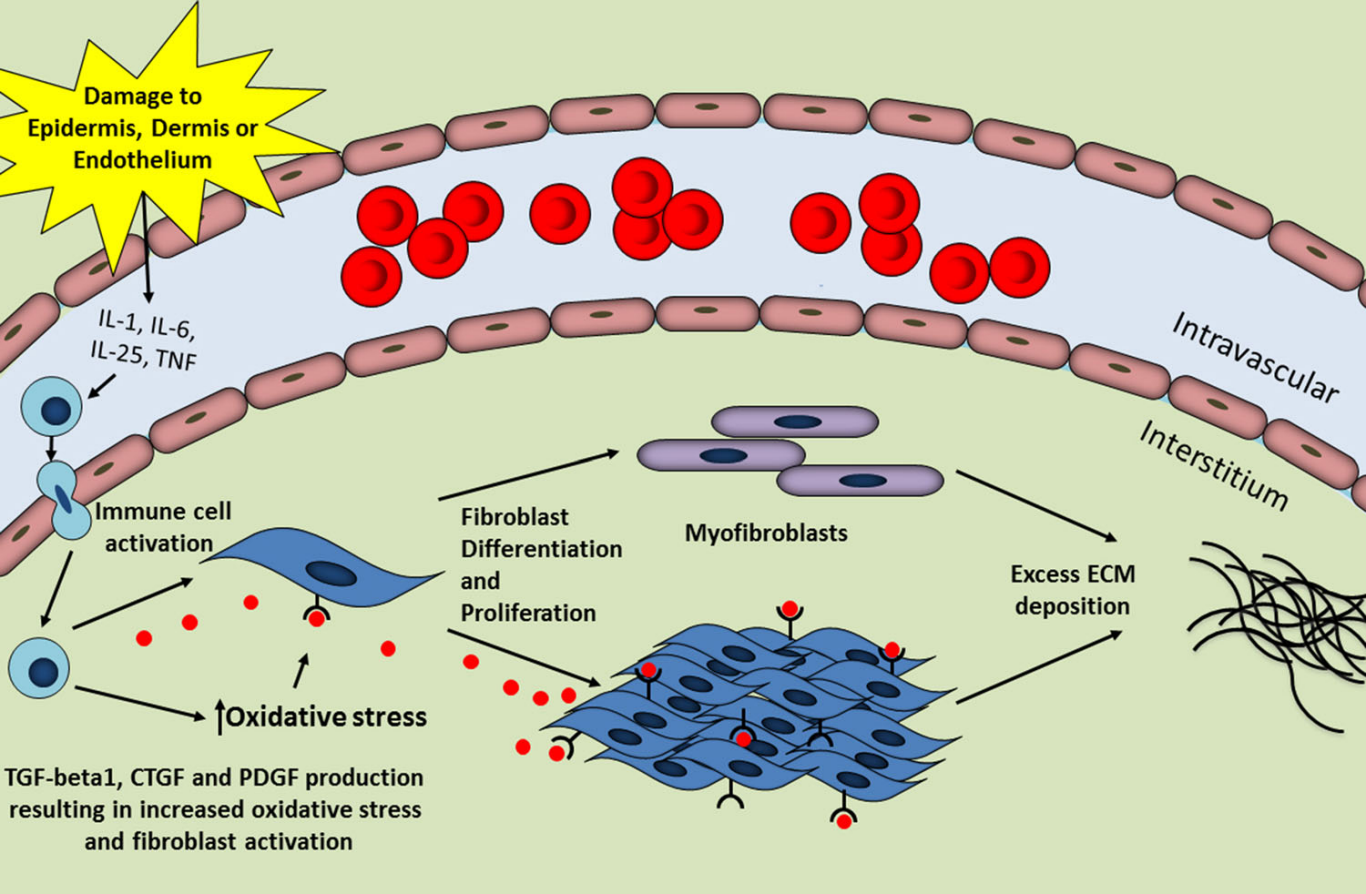
Mechanism of Fibrosis: Damaged endothelial cells secrete chemokines that attract various immune cells. In conjunction with mediators such as reactive oxygen species and other free radicals, these immune cells increase levels of profibrotic growth factors (TGF-beta, PDGF, and CTCF). These growth factors activate fibroblast proliferation and differentiation into myofibroblasts eventually increasing ECM deposition

A summary of the general mechanism of fibrosis is depicted in Fig. 1. Damaged cells recruit multiple regulators of fibrosis such as cytokines, chemokines, angiogenic factors, growth factors, acute phase proteins, and caspases [8]. These regulators recruit endogenous cells such as neutrophils, macrophages, T- and B-lymphocytes that release profibrotic growth factors such as transforming growth factor beta (TGF-beta), connective tissue growth factor (CTGF/CCN2), and platelet-derived growth factor (PDGF). The activation of these agents causes synthesis of ECM via activation and differentiation of myofibroblasts from mesenchymal, epithelial, endothelial, and fibroblast-like cells [8, 11]. In addition to the increased synthesis and deposition of ECM, alpha-smooth muscle actin (α-SMA), a myofibroblast-associated protein which serves to increase contraction of the matrix, and other profibrotic genes are expressed [11]. The progression of fibrosis is a complex process in which oxidative stress is required for progression.

Oxidative stress is thought to play a significant role in cellular functions involved in skin fibrosis [12]. These molecules are defined as free radicals and other unstable oxygen- and nitrogen-containing molecules which have one or more unpaired electrons allowing the formation of other radicals [13]. Exogenously, free radicals are generated by environmental factors including ultraviolet radiation, pollution, cigarette smoke, and other inflammatory processes [3••]. Endogenously, free radicals are formed by enzyme systems including the electron transport chain activity [3••], xanthine oxidase [14], the mitochondrial respiratory chain [15], lipid peroxidases [16], cytochrome P450 [17], NO synthase [18], and multicomponent nicotinamide adenine dinucleotide phosphate (NADPH) oxidases [19•].

## NAPDH Oxidase (NOX) Enzymes

Of interest in fibrosis, NADPH oxidase (Nox) enzyme complexes [20] are located in different cellular tissues and are large producers of oxidative stress [3••, 19•]. In humans, seven Nox enzyme complexes have been identified [20] and have been shown to play a major role in generating radicals such as the superoxide anion, hydrogen peroxide, and singlet oxygen [13, 19•, 20]. Secondary reactive species result from the reactions of free radicals with bystander molecules, proteins, lipids, and nucleic acids [12]. These secondary reactions delineate oxidative stress as both anti-pathogenic and cell signaling molecules.

Oxidative stress has a commonly known protective role during phagocytosis of pathogens [21]; phagocytes are activated to increase free radical production via immunoglobulins, complement proteins, inflammatory cytokines, and different types of activated receptor classes [13]. After phagocyte-mediated effects, free radicals interact with antioxidants such as vitamins, glutathione, or proteins for inactivation [13]. Enzymes including superoxide dismutase (SOD), catalase, glutathione peroxidase, and peroxiredoxins also decrease oxidative stress [13]. Although the link between phagocyte radical production and fibrosis is not well defined, we anticipate this is an important area of future research.

Extracellular signaling is necessary to augment oxidative stress to facilitate their interaction with proteins, DNA, lipids, and carbohydrates. These signals act via three main intracellular signaling pathways, which cause an intracellular increase in oxidative stress along with other downstream actions. These pathways include inhibition of protein tyrosine phosphatases (PTP), activation of MAPK cascades or other kinases, and activation of specific transcription factors [22••].

Interaction of cytokines or growth factors with cell receptors allows for augmentation of oxidative effects. Specific cytokines and signaling molecules such as angiotensin II, PDGF, and TGF-beta have been implicated for their pro-oxidative roles [23]. Once Nox1 and Nox2 are activated via PDGF or Nox4 via TGF-beta, oxidative stress is increased and ECM protein synthesis is modulated via subsequent activation of PTP’s and activation of specific kinases such as JNK, MAPKs, JAKs, c-Src, or extracellular signal-regulated kinase (ERK) [3••]. This results in signal cascade phosphorylation and increased expression of transcription factors as well as fibrotic genes that increase expression of TGF-beta, CTGF (CCN2), and PDGF [1••, 24•]. Thus, oxidative stress has been shown to stimulate expression and secretion of these cytokines and growth factors, influencing differentiation of fibroblasts [25], profibrotic actions [26], and epithelial–mesenchymal transition (EMT) [27]. Additionally, oxidative stress has been associated with aging [28], fibrosis, and scar formation [13, 29–31]. Hence, oxidative stress is necessary for various cellular functions.

Oxidative stress acts to influence cellular fibrosis via a multitude of different cellular signaling actions within the cell. For instance, oxidative stress has been shown to affect profibrotic cytokines and pathways such as TGF-beta and mTOR, cell cycle regulation, EMT, and collagen production. Understanding the mechanisms of oxidative stress interaction with cellular fibrotic actors allows for recognition of additional anti-fibrotic therapeutic targets.

## Oxidative Stress and TGF-beta

Oxidative stress is also influenced by TGF-beta. TGF-beta, a profibrotic cytokine, has been shown to regulate cellular proliferation, differentiation, and ECM production. Research demonstrates that TGF-beta activates NADPH oxidases such as Nox4, augmenting fibroblast recruitment and differentiation [1••, 25, 32, 33•]. Increased Nox expression augments free radical levels, activates the mitochondrial respiratory chain, and represses cellular antioxidant systems [1••]. TGF-beta binding to cell surface receptors causing phosphorylation of Smad transcription factors allows for gene expression of histone acetyltransferases [34].

Mitochondrial free radicals, also increased by TGF-beta signaling, are necessary for TGF-beta-mediated gene transcription in the Smad3 pathways [34]. Within the Smad3 pathways, plasminogen activator inhibitor-1 (PAI-1), a potent profibrotic matricellular protein, is important in TGF-beta1 signaling and is involved in inflammatory and fibrotic pathways [1••, 35–40] via suppression of ECM degradation and increased matrix remodeling [1••, 37]. TGF-beta1 also acts via free radicals on non-Smad pathways including the c-Src-EGFR-MEK-ERK cascade. Smad and non-Smad pathways are influenced by oxidative stress within the cell and interact to increase PAI-1 expression. These pathways suggest a positive feedback loop [34] and highlight the role of oxidative stress in fibrosis. Overall, TGF-beta1 is responsible for activation of multiple oxidative stress-related genes involved in profibrotic pathways [1••, 41–44].

## Oxidative Stress and the Cell Cycle

Free radicals modulate G0, G1, S, G2, and M phases of the cell cycle [45•]. Cells can be influenced to transition from the quiescent (G0) to proliferative (G1, S, G2, and M) stages by free radicals acting as signaling molecules [45•]. For example, a temporary increase in pro-oxidant activity has been shown to be required in mammalian cells during the transition from G1 to S phase [45•]. Antioxidants, such as *N*-acetyl-l-cysteine (NAC) and caffeine inhibit this progression [46, 47]. Additionally, increased SOD activity promotes proliferation with increased hydrogen peroxide activity promoting quiescence in a purported mitochondrial “ROS switch” [45•, 48]. Finally, redox regulation is thought to influence cell cycle proteins such as p21, Rb, cyclin D1/CDK4-6 kinase, and CDC25 [45•].

In fibroblasts, mild elevations in free radicals have been shown to increase proliferation [45•]. However, we have also found that slight increases in oxidative stress are associated with reduced cell counts (unpublished data). Oxidative stress influences insulin-like growth factors (IGFs), AKT/PKB, and phosphoinositide 3-kinase (PI3K) pathways to activate mammalian target of rapamycin (mTOR), which is purported to be responsible for continued translation and cell growth. Influencing continued translation causes mitochondrial oxidative phosphorylation augmenting free radical production within the cell, inherently linking mTOR and free radical generation [49]. As outlined above, oxidative stress effects can be variable with seemingly antagonistic actions on both cell cycle progression and inhibition. Oxidative signaling effects on cellular apoptosis further illustrate this antagonistic phenomenon.

## Oxidative Signaling and Apoptosis

Oxidative stress plays a role in regulating apoptotic proteins and pathways. P53, a tumor suppressing protein demonstrated to be sensitive to free radicals, is important in cell cycle and apoptosis regulation [50]. Generally, stressed cells are inhibited from proceeding through cellular checkpoints. These checkpoints are regulated by p53 which remains activated if cells cannot repair the causative cellular damage. During cellular stress, free radicals can trigger p53 to induce apoptosis. Finally, p53 downregulates manganese superoxide dismutase (MnSOD), an antioxidant enzyme, thereby increasing oxidative stress [51]. Additionally, apoptosis signal-regulating kinase-1 (ASK-1), modulates apoptosis and is a serine/threonine protein kinase causing activation of the p38 and JNK pathways [52–54]. Oxidative stress inhibits caspases and proteases involved in cell death [23].

The counterintuitive roles of oxidative stress in both cell cycle progression and apoptosis seem to present a quandary in terms of understanding cellular responses to oxidative signaling. In actuality, cell cycle signaling initiation and cellular responses to this signaling are complex processes dependent on the free radical cellular levels, with lethal doses signaling for apoptosis while smaller amounts needed to maintain cellular function. Moreover, oxidative changes are also influenced by a myriad of other extracellular and intracellular signals and pathways all co-interacting and enforcing the cell as a complex and dynamic environment.

## Oxidative Stress and Fibroblast Differentiation

The epithelial to mesenchymal transition (EMT) of fibroblasts is required for ECM deposition. Extracellular signals can be generated by various molecules and proteins including collagen, PDGF, TGF-beta, and fibroblast growth factor (FGF) to initiate EMT [22••]. Oxidative stress influences this transition in fibrotic diseases of the lung and kidney [55–57]. Matrix metalloproteinases (MMPs) exposure increases oxidative stress also stimulating differentiation [56]. Differentiated cells, called myofibroblasts, originate from fibroblasts and facilitate wound repair, smooth muscle cell actin expression, and growth factor secretion [24•, 58–60]. With chronic Nox activation and dysregulation of related signaling pathways, activated myofibroblasts, with TGF-beta signaling, differentiate causing a chronic fibrotic state. Further regulation of myofibroblast differentiation is thought to occur secondary to potential interplay between NO/cGMP and Nox4-derived free radicals. Moreover, oxidative signaling with growth factors such as TGF-beta can potentially cause de-differentiation of myofibroblasts into fibroblasts [24•]. Hence, oxidative stress induces myofibroblast differentiation and seems to play a regulatory role.

## Oxidative Stress and Cellular Migration and Adhesion

Oxidative stress plays a large role in fibroblastic migration to sites of inflammation and wound healing. Via oxidative stress, Nox4 has been particularly noted to influence cellular migration [61]. Cellular oxidative stress has been shown to increase IKK (IkB kinase)/NF-κB and JNK/AP-1 (activator protein 1) signaling with consequences on fibroblast migration in cardiac tissue [62]. Increased adhesion is also required for fibrosis. Free radicals are implicated as signaling molecules after integrin attachment during fibroblast adhesion and spreading [63]. Integrins, transmembrane receptors that mediate cell-to-cell attachment within the ECM, have been shown in some studies to increase oxidative stress [63].

## Oxidative Stress and Collagen Production

Increased oxidative stress influences collagen production, a key feature of skin fibrosis. Integrins also mediate collagen production via free radical modulated pathways. Signaling by integrinβ1 activates FAK, allowing for downstream activation of the rac1 protein leading to increased production of collagen and other profibrotic actors such as CTGF(CCN2) and αSMA [64]. CTGF(CCN2) is induced by TGF-beta1 to promote adhesion and fibrosis [65]. Oxidative stress also causes inhibition of the “cysteine switch” which modulates MMPs, proteins responsible for ECM degradation [23, 66, 67]. Hence, studies show oxidative stress plays an essential role in the pathogenesis of ECM deposition including initiation, perpetuation, and regulation of fibrosis.

## Oxidative Stress in Specific Skin Diseases

As outlined above, oxidative stress-mediated actions allow for excessive collagen deposition, typifying the fibrotic phenotype. Our understanding of oxidative stress and its role in cellular signaling in fibrosis continues to expand as studies further delineate their impact on fibrotic disease with mechanisms and findings discussed in the remainder of the paper.

## Graft-Versus-Host Disease

Chronic GVHD is a scleroderma-like disease occurring 2–3 months [68] after allogeneic hemopoietic stem cell transplant. Studies have highlighted the role of CD4^+^ T cells and plasmacytoid dendritic cells in producing free radicals which likely play a large role in GVHD development [69]. Free radicals increase CCL2, a ligand protein previously detected in skin fibroblasts, attracting monocytes and T lymphocytes and elevating collagen, MMP-1, and MMP-2 expression [70]. Additionally, autoantibodies may stimulate free radical production via phosphorylation of the PDGFR tyrosine receptor [3••, 71] and an increase in type I collagen gene expression [69, 71]. Though the role of these autoantibodies has not been fully elucidated, research on this topic continues. Additionally, the therapeutic potential of arsenic trioxide, a trivalent salt which causes hydrogen peroxide toxicity and depletes inherent glutathione levels, showed decreased CD4^+^ T and dendritic cells. This was seen to ameliorate symptomatology in murine mice; skin and visceral fibrosis as well as other autoimmune manifestations were greatly improved [69]. Finally, the organotelluride catalyst, (PHTE) [2] NQ, has been shown to have anti-fibrotic effects via a cytotoxic oxidative pathway in fibroblasts [72]. These studies help to clarify the role of oxidative stress and highlight possible therapeutic modalities which deplete inherent antioxidants and allow accumulation of lethal levels of oxidative stress to target specific disease-associated cells.

## Hypertrophic Scars

Hypertrophic scars are characterized by increased collagen deposition usually caused by injuries in the deep dermis [3••, 73]. They are associated with a change in collagen cross-linking behavior leading to pyridinoline cross-link formation associated with hydroxylysine pathway rather than the usual allysine cross-link formation. This switch has been associated with increased oxidative stress in ex vivo human tissue specimens [74], in which pyridinoline cross-links were also demonstrated to be present in greater amounts in normal skin subjected to artificial free radical generating systems. Cross-links were also seen to be increased in hypertrophic tissues. These findings suggest free radicals may be linked to more pyridinoline in hypertrophic tissue [74]. Additionally, other studies have shown the association between oxidative stress and the apoptosis associated protein, p53, with an interest in increasing apoptotic signaling in myofibroblasts to decrease scar formation [75]. Finally, studies have examined treatment options which target oxidative stress in efforts to decrease scarring [73]. For example, a recent study of essential oil (EO) from rhizomes of *Ligusticum chuanxiong* showed increased free radical production and decreased MMP [76] in human dermal fibroblasts which induced apoptosis. Another compound with positive effects on wound healing includes curcumin, a polyphenol [77, 78]. Curcumin was able to induce apoptosis in human dermal fibroblasts and inhibit fibroblast-mediated contraction, all via oxidative pathways [79]. In vitro findings demonstrate that resveratrol is capable of inhibiting fibroblast function, and it may prove effective in the treatment of hypertrophic scars or keloids in vivo [80, 81]. These compounds call for further research into the potential oxidative-associated clinical sequelae.

## Nephrogenic Systemic Fibrosis

Nephrogenic systemic fibrosis (NSF) is characterized by increased skin fibrosis subsequent to magnetic resonance imaging contrast agents in patients with renal impairment [3••]. This potentially fatal condition usually presents with joint stiffness, tightness, swelling, pain, and joint contraction with limited treatment options [82, 83]. A recent study in murine models has demonstrated free radical involvement in the pathophysiology of this disorder. Additionally, increased Nox4 expression, secondary to TGF-beta1 signaling, led to increased ECM deposition. This study demonstrates a new oxidative therapeutic target for potential treatment options.

## Systemic Sclerosis and Scleroderma

Scleroderma is a connective tissue disorder with immunological, vascular, and fibrotic skin/organ sequelae secondary to increased ECM deposition. At the cellular level, much of the pathogenesis is thought to be due to increased oxidative stress [84]. Free radical production is thought to be secondary to ischemic–reperfusion injury, generation via fibroblasts and leukocytes by the Nox system, and impaired NO metabolism [85–90].

Recent studies show various new signaling pathways in scleroderma including those linking free radicals, Ras, and ERK1–2 which increased the expression of cytokines, growth factors, and their receptors [23]. One study of dermal fibroblasts from scleroderma patients asserted decreased ECM deposition and contraction after fibroblast exposure to antioxidants with oxidative stress suppression, ERK1–2, and NF-kB activity [23, 91]. Understanding these pathways helps to elucidate possible targets for future treatments. Other articles focus on new uses of existing medications such as simvastatin or propylthiouracil, which have been tentatively shown to prevent skin fibrosis and myofibroblast differentiation [92, 93]. Newer studies have also purported arsenic trioxide and (PHTE)(2)NQ, an organotelluride catalyst, which has been shown to prevent both skin and lung fibrosis in murine models of scleroderma via cytotoxic effects on fibroblasts [72, 94], further strengthening the role of oxidative stress in the pathogenesis and therapy of skin fibrosis.

## Therapeutic Intervention in Skin Fibrosis

Oxidative stress can be harnessed for anti-fibrotic therapy by: (1) suppressing free radical production and hindering fibrotic pathways or (2) stimulating oxidative pathways to alter biologic function or reach lethal levels and induce cellular apoptosis. As outlined in Table 2, compounds such as NAC and edaravone execute their effects via the former mechanism with reductions in oxidative stress effects [95, 96]. Conversely, compounds such as EO, arsenic trioxide, and PHTE use the latter mechanism and promote apoptosis via cytotoxicity. Interestingly, some compounds such as trivalent chromium only partially use these methods to decrease fibrosis with activation of caspase-3 via oxidative stress-related apoptotic pathways seen only on initial exposure to the compound with a later switch to cellular necrosis pathways [97]. Finally, concomitant use of both of these mechanisms may fine-tune therapy. For example, curcumin, a polyphenol, was used to induce apoptosis in fibroblasts with antioxidants such as NAC used to moderate its effects [79]. In addition to these compounds, we discuss here the additional studies supporting the use of oxidative stress-associated therapeutics.

**Table 2:** Summary of oxidative stress-associated therapies

Oxidative stress mediating therapeutics	
Oxidative stress-mediated anti-fibrotic therapeutics	Mechanism of action
Simvastatin, propylthiouracil	Prevention of skin fibrosis and myofibroblast differentiation
Arsenic trioxide	Fibroblast cytotoxicity via increased oxidative stress such as H_2_O_2_, depletion of glutathione
(PHTE)(2)NQ	Fibroblast cytotoxicity via increased oxidative stress
Essential oil (EO) from rhizomes of Ligusticum chuanxiong	Increased oxidative stress, increased caspase-3 activity, & decreased MMP all inducing apoptosis
Curcumin	Oxidative stress-related fibroblast apoptosis and inhibition of fibroblast-mediated contraction
*N*-acetyl-l-cysteine	Increased SOD activity causing decreased oxidative stress
Edaravone	Attenuation of fibrotic proteins and cytokines such as interleukin-6 & TGF-b1
Alpha-MSH	Increased SOD2 expression causing decreased oxidative stress
Trivalent chromium	Initial activation of caspase-3 with oxidative stress-related apoptosis pathways with subsequent cellular necrosis pathways
2-deoxy-d-glucose, rapamycin	Decreased oxidative DNA stress-associated proteins
Berberine, vitamin D3, & aspirin	Decreased oxidative stress
Berberine & metformin	Suppression of mTOR signaling
Celecoxib	Antioxidant activity via targeting of DNA oxidative damage
3-bromopyruvate	Suppression of normal cellular metabolic activity and oxidative phosphorylation via inhibition of glycolysis
Hyaluronate	Protective effects on oxidative DNA damage
BMP-7 & rapamycin	Targeting of fibroblastic EMT
Irbesartan	Reduced fibrosis via effects on collagen synthesis

Previous studies on antioxidants such as vitamins C and E have shown little therapeutic impact on skin disorders such as scleroderma [3••, 98, 99]. In addition to NAC, edaravone, and EO as discussed above, studies have suggested a variety of compounds such as berberine, Vitamin D3, and resveratrol, and aspirin effective at reducing oxidative stress [49]. The pathways by which these compounds decrease oxidative stress and attenuate oxidative damage warrant further investigation.

More targeted approaches to treatment on specific signaling pathways have also been proposed. For example, berberine, a natural alkaloid used in Chinese and Ayurvedic medicine, has been shown to suppress mTOR signaling along with metformin, the commonly used diabetic treatment [49]. Fibroblastic EMT has also been suggested as a potential therapeutic target with antioxidants such as BMP-7, rapamycin, GDP, and TGF-beta1 as potential mediators [1••, 100].

Targeting gene expression downstream of the TGF-beta1 signaling pathway could also be an option. Genes which are known to be targets of oxidative stress with downstream profibrotic consequences such as p53, EGFR, Nox4, and Smad3 are also of interest [1••]. Additionally, oxidative stress-dependent genes such as PAI-1, CCN2(CTGF), angiotensinogen, and TGF-beta1 could be used to decrease downstream ECM molecules and other associated genes [1••]. Other potential therapeutic agents which have been shown to reduce lung and kidney fibrosis are ebselen, apocynin, and DPI in murine models [1••]. In fact, simvastatin has been purported to reduce skin thickness along with its beneficial effects on pulmonary fibrosis [92]. Finally, arsenic trioxide has been asserted to improve both skin and lung fibrosis via oxidative stress-mediated killing of activated fibroblasts.

Interestingly, multiple studies in scleroderma, hypertrophic scars, and chronic GVHD have highlighted the therapeutic potential of pro-oxidative compounds. These compounds increase oxidative stress to toxic levels causing cytotoxicity and apoptosis to occur in fibroblasts. Hence, fibrotic activity is controlled and decreased. However, there may be benefit with combined treatments of antioxidants with other therapeutic modalities.

## Conclusion

Oxidative stress has classically been known to play a major regulatory role against pathogens in the phagocytic environment. Recent studies have shown free radicals to play an important role in skin fibrosis. Continued interest in oxidative stress and its processes is necessary to fully elucidate and better treat fibrosis in fibrotic disorders such as scleroderma, GVHD, hypertrophic scars, NSF, and other skin pathologies. Antioxidant therapy continues to be of interest along with new indications for well-known medications. We believe that these and other disorders would greatly benefit from further investigation into oxidative stress signaling pathways and their role in fibrosis, with the potential to transform treatment of skin fibrosis by dermatologists.
